# Frequency reconfigurable open-slot antenna for LTE smartphone applications

**DOI:** 10.1038/s41598-026-49763-x

**Published:** 2026-05-09

**Authors:** Ahmad H. Abdelgwad

**Affiliations:** Department of Electrical Engineering, Fayoum University, Fayoum, 63514 Egypt

**Keywords:** Aperture tuning, Handheld devices, LTE, Open slot, Reconfigurable antenna, Energy science and technology, Engineering

## Abstract

This study introduces a new design for a reconfigurable open-slot antenna tailored for LTE smartphone applications. The antenna is engineered to efficiently cover two broad frequency ranges: 698–960 MHz and 1710–2690 MHz. To minimize the used space, the slot is positioned at the top of the smartphone’s printed circuit board (PCB) and by incorporating switches loaded with lumped components, the design effectively tunes into the lower frequency bands while keeping the antenna compact. The working mechanism of the antenna was carefully analyzed, with the final version optimized, fabricated, and tested. Simulated and measured results show that the antenna consistently provides a great performance across all target frequency bands. This makes it a strong and practical solution for modern LTE smartphones and similar handheld devices.

## Introduction

Recently, the rapid evolution of wireless communication systems has led to the deployment of numerous standards operating across diverse frequency ranges and protocols worldwide^1^. To ensure compatibility with these systems, mobile handset antennas are required to support operation across multiple frequency bands. Concurrently, the trend toward larger displays with minimal bezels in modern smartphones has significantly reduced the available space for antenna integration^2^. Consequently, the design of compact multiband or wideband antennas has emerged as both a critical requirement and a substantial technical challenge. These antennas must reliably operate within the 698–960 MHz and 1710–2690 MHz frequency ranges, encompassing widely used bands such as LTE700, GSM850, GSM900, GSM1800, GSM1900, UMTS, LTE2300, and LTE2500. Additionally, a minimum total efficiency of 50% across these bands is typically required to ensure adequate performance. Over the past two decades, numerous antenna configurations have been proposed in the literature to address these demands^3–6^. These include single- and dual-band planar inverted-F antennas (PIFAs), capacitive and shorting techniques^7^, capacitive feeding methods^8^, the integration of parasitic elements^9^, resonant slot structures^10^, loop antennas^11^, and active antenna designs^12^. Each of these approaches contributes unique advantages toward achieving compact, high-efficiency antenna solutions suitable for contemporary mobile devices^13^.

Compact, wideband or multiband antenna design for smartphones remains a significant challenge due to the severe space constraints. Conventional compact internal antennas, such as planar inverted-F antennas (PIFAs), monopoles, and loops, often struggle to deliver wideband performance or multi-band coverage^14^. Therefore, various strategies have been explored to overcome this limitation in mobile phone applications. This includes using multiple slots to induce the necessary resonances for low band coverage^15^. In another attempt, an IFA combined with a slot in the ground plane enabled operation across GSM850/900, DCS, PCS, UMTS2100, and LTE2300/2500 bands^16^. Additional designs for laptops and tablets incorporated open-slot antennas with dual-feed configurations^17^ or large ground clearance^18^. A more recent approach is to utilize varactor diodes or PIN diodes for antenna state reconfiguration^19–34^, offering a promising direction, albeit with partial coverage of the lower frequency band. However, many of the proposed solutions either required substantial space, complex to be implemented, required many switches or failed to cover all the necessary frequency bands.

In this context, to address these limitations, this work proposes a reconfigurable open-slot antenna excited by an L-shaped microstrip feed. This design supports octa-band operation while maintaining a compact footprint. By stimulating the antenna’s fundamental mode, the design achieves effective miniaturization and can be easily integrated into standard smartphone printed circuit boards (PCBs) with small clearance area. Reconfiguration is achieved via the slot aperture turning using switches loaded with lumped components in series, enabling multiple operational states. As a result, the proposed antenna successfully covers the full LTE low, mid and high bands, from 698 to 960 MHz and 1710–2690 MHz. The proposed design distinguishes itself by integrating a capacitor and an inductor in a compact reconfigurable structure with three switching states, enabling seamless tuning across multiple operating states while maintaining stable performance. Unlike conventional slot or previously presented reconfigurable solutions that often require larger ground clearance, more complexity, or compromise efficiency, this antenna achieves wide coverage of LTE bands with a simple design, minimal footprint and consistent high efficiency. This balance of compactness, broadband operation, and reliable reconfigurability highlights the antenna’s suitability for next-generation smartphones where space optimization and multi-band compatibility are critical.

The structure of this paper is as follows: Sect. 2 details the antenna design, operation and prototype implementation. Section 3 discusses both the simulated and experimental results, including S-parameters, antenna efficiency, and radiation patterns. The proposed antenna performance is compared relative to recently reported reconfigurable designs in Sect. 4. Finally, Sect. 5 concludes the paper by summarizing the key findings.

## Reconfigurable Slot Antenna Design and Operation

**Fig. 1:**
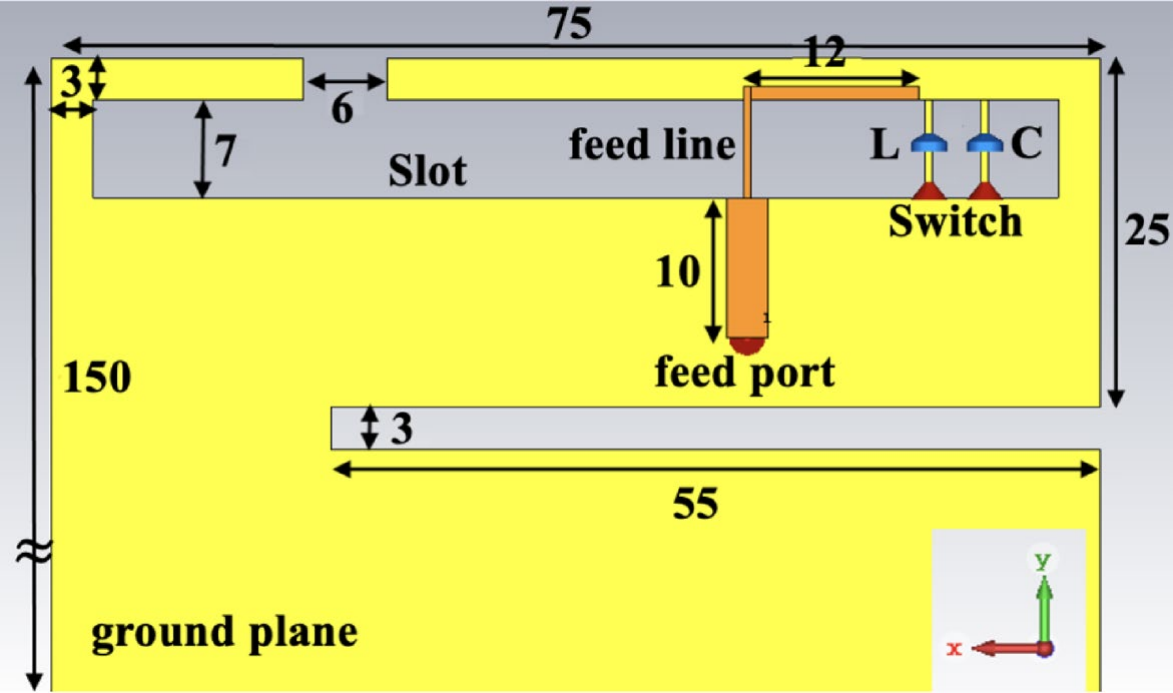
Proposed reconfigurable slot antenna design.

A reconfigurable open-slot antenna design tailored for LTE smartphone applications is proposed and the configuration of the antenna is depicted in Fig. 1, where the antenna is integrated on a system circuit board with dimensions of 150 × 75 mm², fabricated on an FR4 substrate (ε_r_ = 4.4, tanδ = 0.02) with a thickness of 0.8 mm. The structure features an asymmetrical inverted T-shaped wide slot etched directly into the circuit board. Opposite this slot, an L-shaped feed strip is positioned on the other side of the substrate, functioning as the primary excitation element. The position and length of the capacitive feed line play a critical role in determining the capacitive loading introduced, which in turn influences the resonant frequencies, increasing the feed length results in increased capacitive loading and lowers the resonance frequency. Additionally, a narrow slit of 3 mm in width and 55 mm in length is introduced into the ground plane. This cut helps manipulate the current distribution on the ground plane and contributes to shifting the resonant frequencies to lower values. To facilitate frequency reconfigurability, the slot is equipped with two switching elements: one connected in series with an inductor (L = 15 nH) and the other with a capacitor (C = 2.5 pF). These components enable dynamic tuning of the slot’s aperture, allowing for adjustments in operational frequency.

**Fig. 2:**
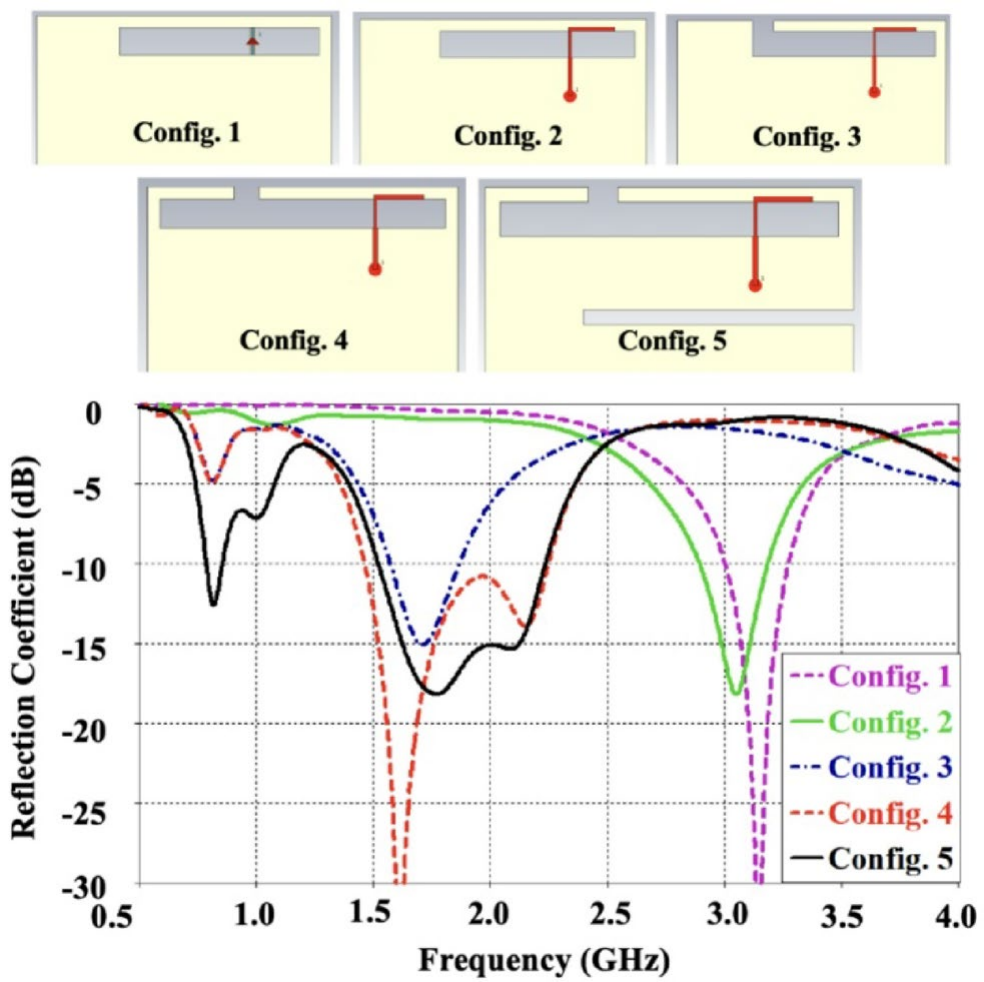
Simulated reflection coefficient (S_11_ in dB) for simple configurations of proposed antenna.

The operating mechanism of the proposed structure can be interpreted through five fundamental antenna configurations illustrated in Fig. 2, where simplified layouts are presented without incorporating reconfiguration switches. A closed-slot antenna is generally known to resonate at approximately 0.5λ. However, when the slot is opened, the resonance shifts to 0.25λ, enabling a more compact design. Furthermore, the use of an FR4 substrate introduces a loading effect that reduces the resonant length of the slot to nearly 0.16λ. As depicted in Fig. 2, Configuration 1 employs a closed slot etched in the ground plane, directly fed to excite the 0.5λ slot mode, yielding a simulated resonance near 3.2 GHz. In Configuration 2, the same closed slot is excited through an L-shaped feed strip, producing a capacitive coupling effect that shifts the resonance slightly lower to around 3.0 GHz. Configuration 3 modifies the design by opening the ground slot while maintaining approximately the same length, resulting in dual resonances at 0.8 GHz and 1.7 GHz. These modes correspond to the 0.16λ slot and 0.25λ inverted-F antenna (IFA) behaviors, respectively, thus enabling operation in the low-frequency region. In Configuration 4, the open-slot length is extended, introducing an additional resonance at about 2.2 GHz due to the 0.25λ slot mode. However, this arrangement does not achieve adequate impedance matching in the low band. To address this limitation, Configuration 5 incorporates a ground slit, which improves impedance characteristics around 0.8 GHz. Afterward, the geometry is optimized, and reconfigurable switches with lumped components are integrated into the design to ensure coverage across the targeted LTE bands.

The proposed antenna (Fig. 1) exhibits a single resonant mode at the lower frequency band and two distinct modes at higher bands. The capacitive feed strip contributes to the overall loading effect and enables fine-tuning of all three resonant frequencies by varying its dimensions and location. In the lower frequency band, resonance is primarily governed by the length of the longer side of the open slot, resulting in operation near 700 MHz. At higher frequencies, two resonances appear: one around 1.8 GHz, attributed to an inverted-F antenna (IFA) mode formed by the slot and feed line, and another near 2.6 GHz due to the shorter side of the slot acting as a 0.25λ open-slot radiator.

To extend coverage to the LTE low band (698–960 MHz), frequency reconfiguration is implemented using the switchable loading elements, either the inductor or capacitor can be engaged via the switches. This approach allows the lower-band resonance to be tuned, offering dynamic control over the antenna’s performance. As a result, the proposed antenna design supports multi-band operation across eight LTE-relevant frequency bands, including LTE700, GSM850/900, DCS, PCS, UMTS2100, and LTE2300/2500.

**Fig. 3:**
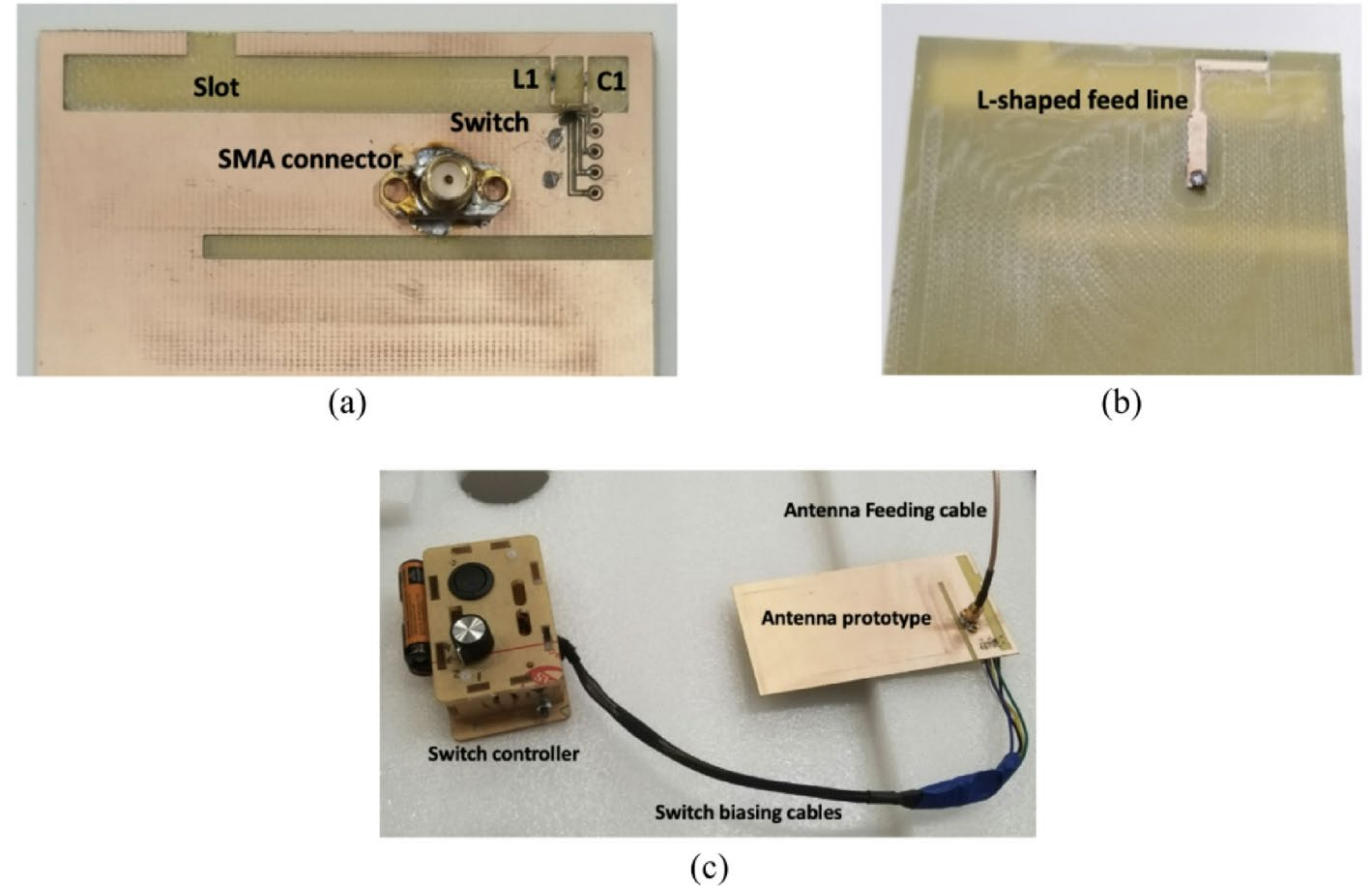
Fabricated prototype **(a)** front view **(b)** back view **(c)** prototype with switch controller.

To validate the proposed reconfigurable open-slot antenna design, a prototype was fabricated and experimentally tested. The completed antenna, along with its integrated biasing network for switch control, is displayed in Fig. 3. The feeding mechanism employs a standard 50-Ω SMA connector for reliable signal input.

**Fig. 4:**
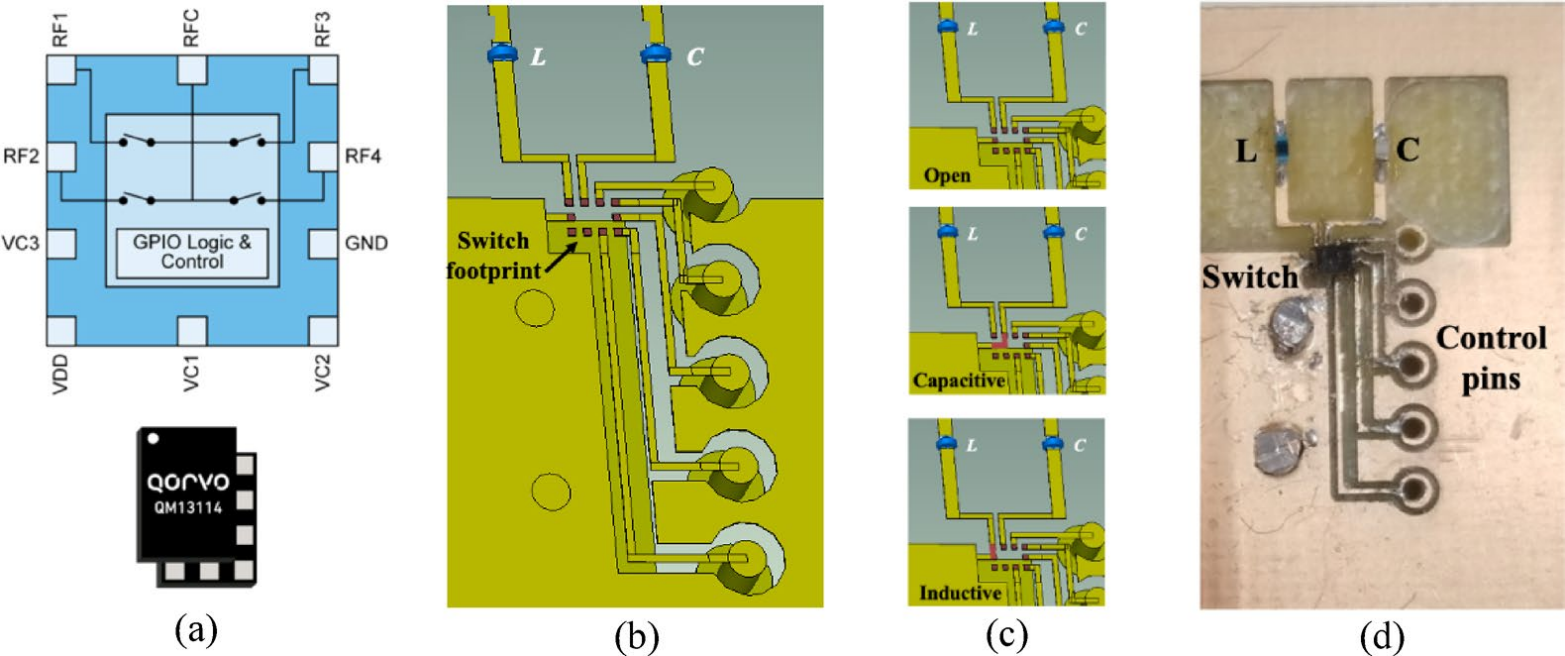
Reconfiguration circuit **(a)** switch functional block diagram **(b)** simulated footprint layout **(c)** emulation of different reconfiguration states **(d)** fabricated prototype with the switch soldered on the footprint.

A Qorvo QM13114 single-pole four-throw (SP4T) RF switch is utilized to alternate between the various lumped loading configurations in the proposed design. The functional block diagram, simulated footprint, and detailed prototype image with the integrated switch are presented in Fig. 4. As illustrated, the switch is integrated into the antenna structure to realize three distinct states—open, capacitive, and inductive loading. The switch is positioned on a designated footprint located near the slot edge on the FR4 substrate. The biasing and control signals are applied through PCB-printed control lines, which are interfaced with the external controller via dedicated biasing cables, as shown in Fig. 3(c).

The insertion losses and parasitic parameters extracted from the manufacturer’s datasheet were incorporated into the full-wave simulation model to accurately account for the switching network’s influence on antenna behavior. A combined electromagnetic and circuit-level co-simulation was conducted to evaluate the impact of the reconfiguration circuitry on antenna performance. The results indicate that the insertion loss of each switching element remains below 0.3 dB across the operational frequency range, while the associated parasitic capacitance and series resistance produce only minor variations in the impedance response. The switch footprint and bias line layout were carefully optimized to suppress unwanted coupling and preserve impedance matching in all reconfiguration states.

Additionally, the effect of the switching network on the antenna’s efficiency and radiation characteristics was investigated. The simulation outcomes reveal a slight efficiency reduction of less than 2%, primarily due to the biasing network, without significant alteration of the radiation patterns or beam reconfigurability. These observations confirm that the adopted switching architecture effectively supports the desired operational versatility with negligible degradation in overall antenna performance.

Biasing of the RF switch is managed via a DC control circuit, with biasing lines connected as illustrated in Fig. 3(c). To demonstrate the antenna’s reconfigurability, three distinct switching configurations were examined:

- **State 1 (Open State)**: The RF switch remains off, isolating both the capacitor and inductor from the slot. This configuration reflects the intrinsic resonance behavior of the unloaded slot antenna.
- **State 2 (Capacitive Loading)**: The capacitor is connected while the inductor remains disconnected. This introduces capacitive loading to the slot, resulting in a downward shift in the resonant frequency.
- **State 3 (Inductive Loading)**: The inductor is connected, and the capacitor is disconnected. This inductive loading causes the antenna’s resonant frequency to shift upward.

## Results and discussions

**Fig. 5:**
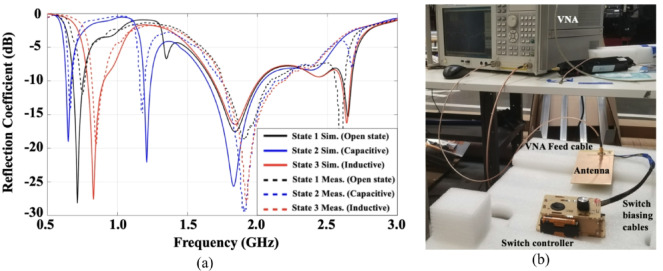
**(a)** Simulated and measured reflection coefficient (S_11_ in dB) of proposed reconfigurable slot antenna for different switch cases **(b)** S-parameters measurement setup.

The simulated and measured reflection coefficients (S_11_) of the proposed reconfigurable open-slot antenna (Fig. 1) are presented in Fig. 5(a). The setup employed for measuring the reflection coefficient using a vector network analyzer (VNA) is illustrated in Fig. 5(b). The antenna prototype is interfaced with the VNA through an SMA connector linked to the L-shaped feeding strip. A switch controller regulates the operating state of the switch—open, capacitive, or inductive—by applying the appropriate DC bias through the control cable. To minimize environmental interference and emulate free-space conditions, the antenna is positioned on a thick foam substrate during the measurement process. The antenna S-parameters demonstrate effective operation across three distinct resonant frequencies, covering the major LTE bands: the low band (698–960 MHz), mid band, and high band (1710–2690 MHz) with return loss better than 6 dB. Among these, achieving adequate performance in the low-frequency band poses the greatest design challenge due to the physical size typically required to support resonance at such long wavelengths.

To address this, the antenna leverages its reconfigurable design, enabling dynamic tuning through the activation of different switch states. When the capacitive loading path is engaged (State 2), the added capacitance lowers the effective electrical length of the resonator, resulting in a downward shift of the low-band resonance. Conversely, inductive loading (State 3) increases the reactance, shifting the resonance upward. The unloaded (open) state (State 1) provides a mid-range resonant frequency, offering a balanced operating point between the two extremes.

This ability to finely control the resonance behavior enables the antenna to adapt its performance and effectively cover the required LTE spectrum without increasing the physical footprint. As shown in Fig. 5(a), there is a strong correlation between the simulated and measured results, which confirms the accuracy of the simulation model and the reliability of the fabricated prototype. These results clearly validate the design concept and demonstrate the efficacy of the reconfiguration mechanism in achieving wideband, multiband operation suitable for modern smartphone applications.

**Fig. 6:**
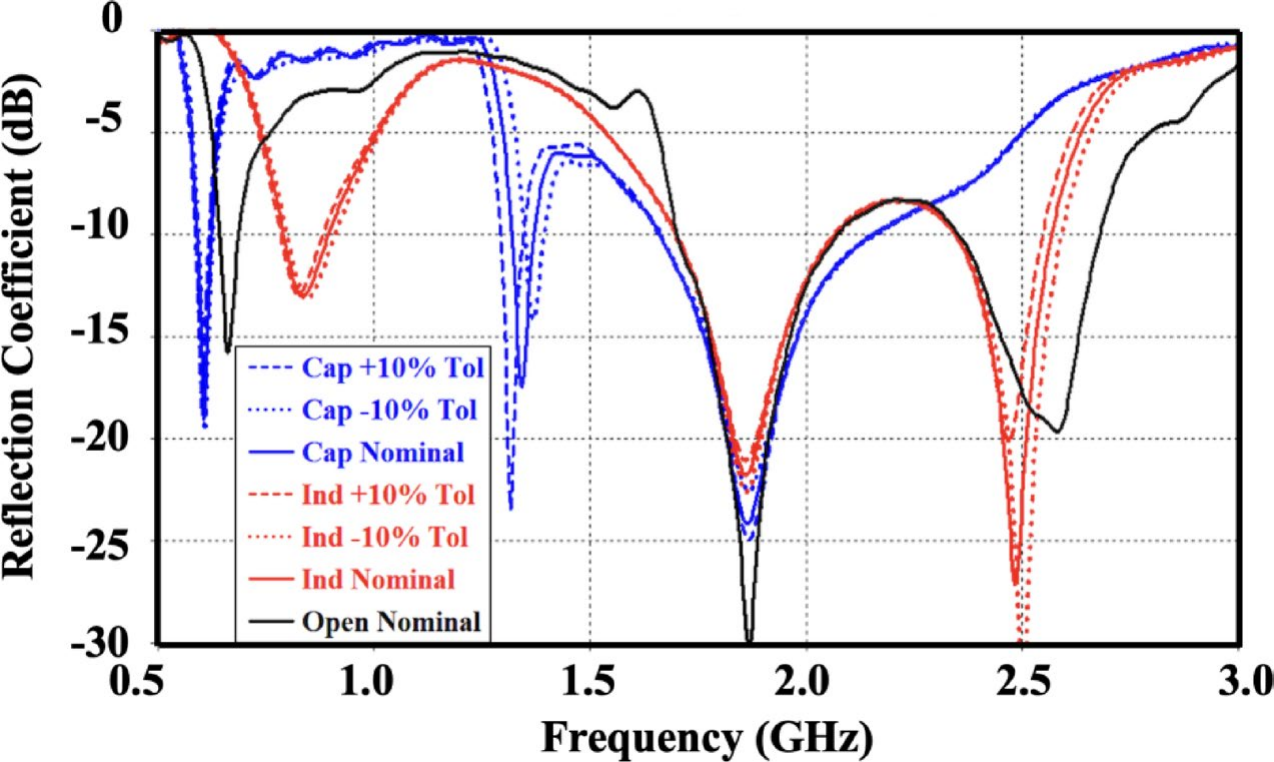
Simulated reflection coefficient (S₁₁ in dB) of the proposed antenna under different switch states, considering a 10% tolerance in the reconfigurable components.

The stability of the proposed antenna across its reconfigurable operating modes was evaluated by introducing a ± 10% variation in the reactive loading elements (L and C), as illustrated in Fig. 6. The simulated results show that such variations lead only to slight resonant frequency displacements, remaining within 3% of the nominal values, without causing performance gaps or inactive regions within the targeted bands. The reflection coefficient responses corresponding to the three switching configurations demonstrate a gradual and overlapping behavior, which guarantees uninterrupted frequency coverage throughout the 0.7–2.7 GHz spectrum. Furthermore, the employed capacitive elements and high-quality-factor inductors are characterized by minimal reactance drift over a wide temperature range from − 20 °C to 80 °C, reinforcing the reliability of the antenna operation. In real-world applications, such as mobile terminals, minor frequency shifts induced by temperature or environmental effects can be effectively mitigated through adaptive matching networks or impedance tuning circuits commonly available in RF front-end architectures. Consequently, the proposed antenna preserves stable and continuous multiband functionality despite component tolerances and thermal variations.

**Fig. 7:**
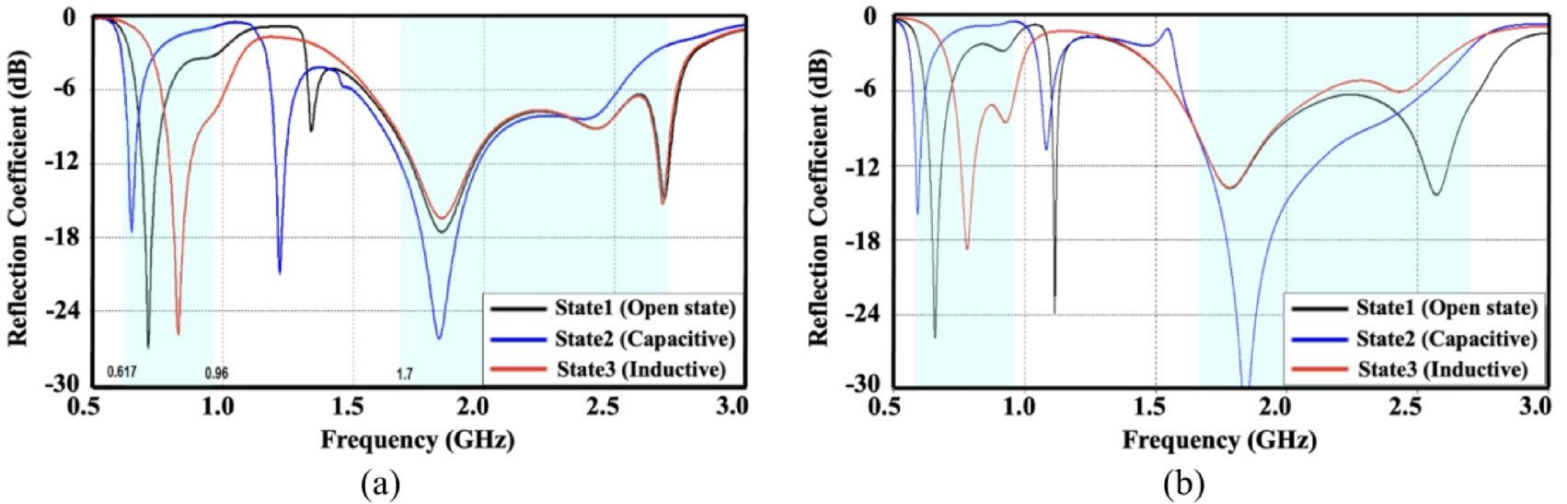
Simulated reflection coefficient (dB) **(a)** Proposed design with ground length 150-mm **(b)** Design with extended ground length (170-mm).

To examine the influence of the ground plane dimensions on the antenna characteristics, the reflection coefficients for two representative smartphone ground plane lengths were analyzed, as shown in Fig. 7. The comparison illustrates the reflection responses corresponding to different reconfiguration states for ground lengths of 150 mm and 170 mm. It is observed that, even with the extended ground plane, the antenna maintains satisfactory impedance matching across the targeted frequency bands of 698–960 MHz and 1710–2690 MHz, confirming the robustness and consistent performance of the proposed design under varying smartphone ground plane conditions.

**Fig. 8:**
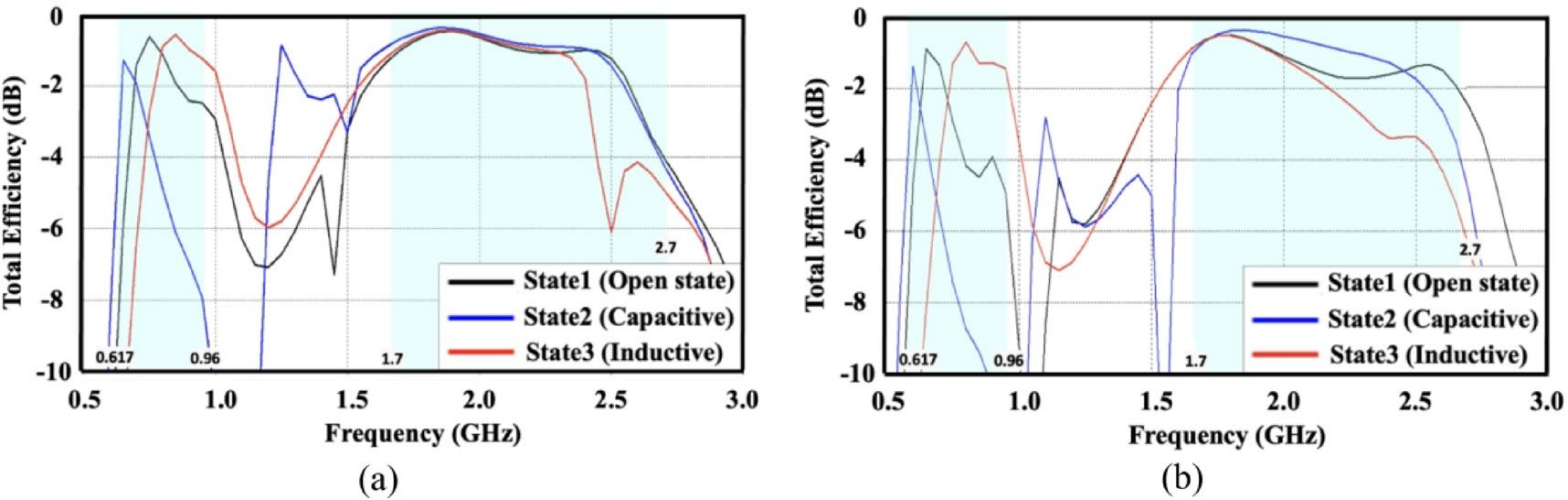
Total antenna efficiency (in dB) for different switch states **(a)** Proposed design with 150-mm ground length **(b)** Design with extended ground length 170-mm.

The total efficiency of the proposed reconfigurable antenna under various switching conditions is presented in Fig. 8(a). For the lower frequency band (617–960 MHz), the antenna consistently maintains a high total efficiency, exceeding − 4 dB, across the three switching states. This stability is attributed to the aperture tuning effect achieved by loading the slot with either an inductor or a capacitor, which effectively shifts the antenna’s resonant frequencies. In the upper band (1710–2690 MHz), the antenna also demonstrates a total efficiency above − 4 dB throughout the mid and upper LTE bands. Overall, the proposed design delivers wideband coverage across the low, mid, and high LTE bands while preserving a compact form factor and high efficiency. Furthermore, to evaluate the influence of ground plane dimensions on the antenna efficiency across different configuration states, Fig. 8(b) presents the simulated efficiency results for the design with an extended ground length of 170 mm. It can be observed that the antenna maintains a stable efficiency level, remaining above − 2 dB throughout the LTE frequency bands of interest, thereby confirming the robustness of the proposed configuration against variations in ground plane size.

**Fig. 9:**
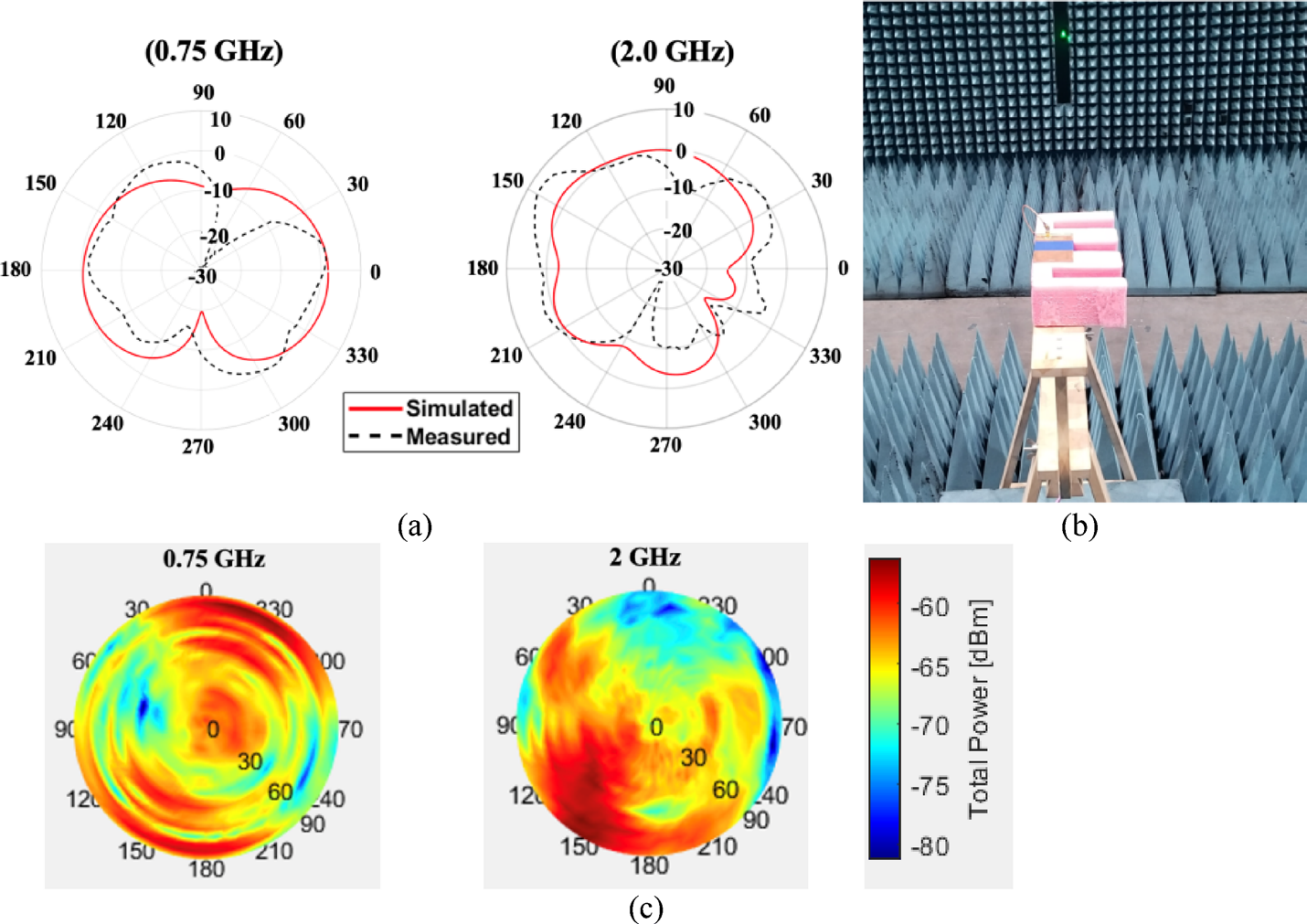
**(a)** Simulated and measured azimuth plane antenna pattern **(b)** OTA measurement setup in the anechoic chamber **(c)** Measured total radiated power distribution (dBm).

The over-the-air (OTA) measurement setup for the proposed frequency-reconfigurable slot antenna was carried out inside an anechoic chamber to ensure a controlled and reflection-free environment. The antenna under test was mounted on a non-conductive positioner at the center of the chamber and aligned toward a standard linearly polarized reference antenna. A vector network analyzer was used to record the radiation characteristics and gain across the target LTE frequency bands, while the reconfigurable states were controlled through an external DC bias circuit. This setup enabled accurate evaluation of the antenna’s free-space far-field performance, including radiation patterns and efficiency, under different operating configurations. Figure 9(a) presents the simulated and measured azimuth plane far-field gain radiation patterns at 750 MHz and 2 GHz, while Fig. 9(b) illustrates the measured total radiated power (TRP) distribution at the same frequencies. As observed, there is a good correlation between the simulated and experimental results, indicating the reliability of the design. The radiation patterns exhibit noticeable asymmetry, which is attributed to the inherently asymmetrical geometry of the antenna structure. Despite this, the patterns maintain stable directional characteristics, validating the antenna’s suitability for practical applications across the targeted frequency bands.

## Performance evaluation

### Antenna performance in free space

Table 1 presents a comprehensive summary of the proposed antenna’s performance in Free space under various reconfigurable states. The results indicate that the antenna maintains effective multiband operation, covering the principal LTE bands—specifically the low band (approximately 700–960 MHz), mid band, and high band (1.5–2.7 GHz)—with reflection coefficients below − 6 dB across all states. The average radiation efficiency varies between 70% and 87%, while the total efficiency remains better than − 2 dB, demonstrating minimal losses introduced by the reconfiguration mechanism. The total radiated power (TRP) remains stable, ranging from 18.2 to 19.0 dBm for a 20 dBm input power, confirming consistent radiation performance across the different loading conditions (open, capacitive, and inductive). These results verify that the antenna design achieves wide impedance bandwidth, satisfactory efficiency, and reliable power radiation characteristics suitable for modern LTE handheld applications.

**Table 1:** Proposed antenna different reconfigurable states free space performance.

State	bandwidth MHz (BW%) (S_11_ < −6 dB)	Average Radiation Efficiency dB (%)	Average Total Efficiency dB (%)	TRP (dBm)
State 1 (Open)	700–810 MHz (15%), 1550–2750 MHz (56%)	−0.8 dB (84%) −0.9 dB (81%)	−1.0 dB (79%) −1.2 dB (76%)	19.0 dBm 18.8 dBm
State 2 (Capacitive loading)	610–720 MHz (17%), 1500–2500 MHz (50%)	−0.6 dB (87%) −0.8 dB (84%)	−1.6 dB (69%) −1.2 dB (76%)	18.4 dBm 18.8 dBm
State 3 (Inductive loading)	790–1000 MHz (23%), 1560–2750 MHz (55%)	−0.8 dB (83%) −1.6 dB (70%)	−1.1 dB (78%) −1.8 dB (66%)	18.9 dBm 18.2 dBm

### Effect of User Proximity and SAR Performance

**Fig. 10:**
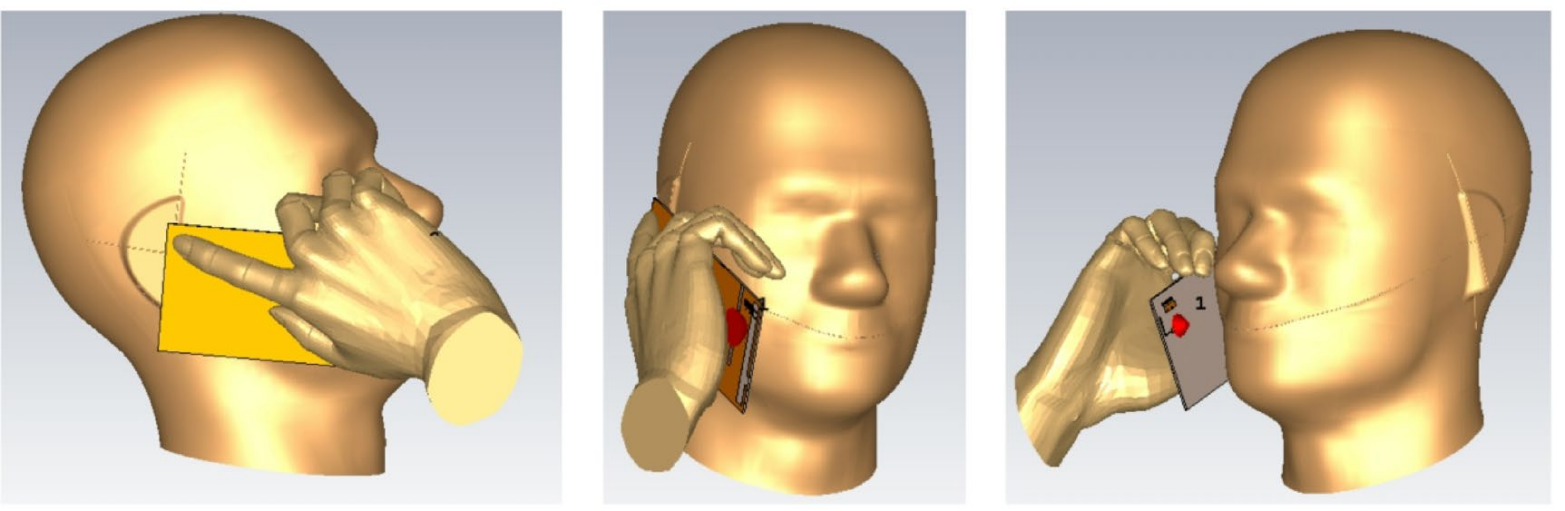
Hand and head SAR simulation model.

The influence of the user’s hand and head on the antenna performance, as well as its specific absorption rate (SAR) compliance, has been thoroughly examined. Figure 10 illustrates the human hand and head phantoms employed for SAR computation and performance assessment in proximity to the antenna. The adopted simulation setup closely resembles the CTIA-recommended models commonly utilized in industry for evaluating device performance under realistic user conditions. Table 2 summarizes the impedance bandwidths obtained for different reconfigurable states when the antenna is placed near the human body, along with the corresponding 1-g averaged SAR values calculated using CST Studio Suite (version 2022)^35^. As expected, a slight frequency detuning is observed due to the loading effect of the body phantoms; however, the antenna maintains sufficient tuning stability to ensure reliable coverage across the targeted LTE bands. For SAR estimation, a transmit power of 24 dBm is applied, and the 1-g average SAR is computed. The resulting values remain well below the regulatory threshold of 1.6 W/kg, confirming compliance with the established safety standards.

**Table 2:** Antenna bandwidth close to head and hand and simulated SAR for 1-g Tissue.

State	bandwidth MHz (BW%) (S_11_ < −6 dB)	1-g SAR (W/Kg), Input Power = 24 dBm							
		0.7 GHz	0.8 GHz	0.9 GHz	1.0 GHz	1.7 GHz	2.0 GHz	2.5 GHz	2.7 GHz
State 1 (Open)	700–980 MHz (%), 1620–2430 MHz (%)	0.50	0.72	0.80	0.53	0.55	0.23	0.61	0.51
State 2 (Capacitive loading)	650–730 MHz (%), 1450–2460 MHz (%)	0.64	0.55	0.21	0.002	0.56	0.50	0.67	0.55
State 3 (Inductive loading)	700–980 MHz (%), 1620–2430 MHz (%)	0.50	0.72	0.80	0.53	0.55	0.23	0.61	0.51

## State-of-the-art comparison

To evaluate the effectiveness of the proposed antenna in comparison with other relative frequency-reconfigurable configurations available in the literature, a detailed performance comparison has been carried out and summarized in Table 3. The analysis benchmarks the proposed structure against previously reported antennas by considering critical design parameters, including overall structural complexity, physical dimensions, switching methodology, achievable impedance bandwidth, and suitability for practical communication applications.

From the comparison, it is evident that the proposed antenna achieves a balanced trade-off between compactness and performance. The antenna employs a simple and low-profile configuration, maintaining a reduced ground clearance suitable for operation in the lower LTE frequency bands, while simultaneously attaining a considerably broader bandwidth than most comparable compact designs.

While previous studies have extensively utilized PIN diodes or varactor elements to realize frequency tunability, the present design introduces a distinctive tuning mechanism in which each switch is combined with either inductive or capacitive loading components to produce three distinct reconfigurable states. This hybrid switching strategy effectively extends the impedance bandwidth through slot aperture tuning, without increasing circuit complexity or introducing excessive insertion loss that could degrade radiation efficiency.

Overall, the comparative evaluation underscores that the proposed antenna achieves an advantageous balance of structural simplicity, wide reconfigurable bandwidth, compact geometry, and broad application compatibility, establishing it as a practical and competitive solution for modern LTE-enabled portable communication systems.

**Table 3:** Comparison of proposed antenna performance with recent frequency reconfigurable antenna designs.

References	Antenna Structure	Footprint (mm^2^)	Switching Device	S_11_ Bandwidth (GHz)	Fractional Bandwidth (%)	Applications
^25^	Multilayered Patch (Complex)	80.3 × 80.3	1 PIN	1.545–1.555, 2.515–2.585, 1.642–1.657, 2.47–2.51	0.64%, 2.55%, 1.81%, 1.6%	GPS, WLAN2.4G Bluetooth
^26^	L-shaped Patch (Simple)	40 × 35	1 PIN	3.6–4.8, 2.1–2.5, 5–5.6, 2.8–3.6, 5.5–6.1	30%, 16.66% 11.3%, 24.24%, 10.16%	WLAN2.4G, Bluetooth 5G Wi-Fi
^27^	Slot Antenna (Complex)	20 × 20	3 PIN	2.3–2.51, 3.35–3.75, 4.95–5.53	8.7%, 11.2%, 11%	WLAN2.4G, Bluetooth 5G Wi-Fi 3.5GHzWiMAX
^28^	Slotted Circular patch (Complex)	100 × 100	4 PIN	2.37–2.67, 3.39–3.62	12.24%, 6.57%	WLAN2.4G, Bluetooth WiMAX
^29^	Pentagonal slots (Simple)	60 × 120	2 Varactors	1.64–2.62	23%	WLAN2.4G, Bluetooth, Cognitive Radio
^30^	Loop Antenna (Complex)	25 × 10	4 Varactors	0.698–0.96, 1.6–2.6, 3.4–3.8, 5.0–6.0	31.6%, 47.6%, 11.1%, 18.1%	LTE700, WLAN2.4G, Bluetooth 5G Wi-Fi 3.5GHzWiMAX
^31^	Dipole Antenna (Complex)	60 × 30.6	8 Varactors	1.98–2.76	32.9%	WLAN2.4G, Bluetooth, LTE2300/2400
^32^	Slotted Monopole (Complex)	88 × 88	8 Varactors	0.76–1.04, 1.50–1.87	31.1% 29.96%	GPS
^33^	Patch with Shorting stubs (Complex)	131.47 × 131.47	9 Varactors	2.22–2.79, 3.15–3.96, 4.23–5.0	22.8%, 22.8%, 16.7%	IOT
^34^	Slot Antenna CPW Feed (Simple)	28 × 59	4 Switches	2.3–2.75	17.8%	IOT
Proposed Antenna	Slot Antenna (Simple)	150 × 75	2 PIN	0.698–0.960, 1.71–2.69	31.6% 44.55%	LTE700, GSM850/900, DCS, PCS, WLAN2.4G, Bluetooth, UMTS2100, LTE2300/2500.

## Conclusion

This work has introduced and examined a compact, reconfigurable open-slot antenna designed to support octa-band operation for smartphone LTE applications. By employing aperture tuning through lumped components, the antenna achieves multiple operating states, maintaining a return loss greater than 6 dB across the frequency ranges of 698–960 MHz and 1710–2690 MHz. The design requires minimal ground clearance, enabling a high level of miniaturization suitable for integration into modern smartphones. The antenna has been successfully designed, prototyped, and tested, with key performance metrics thoroughly analyzed to validate its effectiveness and practicality.

## Data Availability

All data generated or analysed during this study are included in this published article.
